# The influence and spatial spillover effects of producer services agglomeration on urban green development in China

**DOI:** 10.1371/journal.pone.0315870

**Published:** 2024-12-30

**Authors:** Weimin Gong, Chengxin Wang, Dan Men, Ming Zhang, Jian Wang

**Affiliations:** 1 School of Tourism, Shandong Women’s University, Jinan, China; 2 College of Geography and Environment, Shandong Normal University, Jinan, China; 3 College of Geography and Environmental Science, Northwest Normal University Lanzhou, Lanzhou, China; Dongshin University, REPUBLIC OF KOREA

## Abstract

Based on the analysis of the spatiotemporal evolution characteristics of producer services agglomeration and urban green development efficiency in China, this study measures the influence and spatial spillover effects of producer services agglomeration on urban green development. Research results reveal that the specialization agglomeration level of producer services has undergone a dynamic decline process, demonstrating spatial characteristics where the east exhibits higher levels than the west, and the north surpasses the south. In contrast, the diversification agglomeration level of producer services has demonstrated a consistent upward trajectory, characterized by a spatial distribution that is broadly scattered but concentrated on a smaller scale. Regarding China’s urban green development, its efficiency shows a dynamic upward trend and displays characteristics of agglomeration and contiguous development in space. Overall, both modes of producer services agglomeration have beneficial diffusion impacts on urban ecological advancement. Furthermore, the impact of this agglomeration on the efficiency of green development notably varies across regions and industries.

## Introduction

The key role of green development in achieving economic, social and ecological sustainable sustainability is increasingly recognized as a consensus within the international community [1]. China has a long and rich history of sustainable development, which includes the ancient concept of harmony between nature and man, and the modern emphasis on valuing the natural environment and green development, both of which embody the principles of sustainable development [2]. Against the backdrop of advancing energy conservation, emission reduction and high-quality economic development, China’s economy has progressed to a stage focused on enhancing quality and efficiency. Therefore, accelerating the development of a green and low-carbon modern industrial system is crucial for achieving green urban development in China [3]. As an intermediate industry supporting manufacturing and a modern service with agglomeration characteristics, producer services are playing an increasingly important role in driving industrial structure transformation and enhancing the effectiveness of sustainable growth [4, 5]. Consequently, analyzing the impact and spatial effects of producer services agglomeration on urban ecological efficiency is crucial for advancing green development theory and exploring a green development path tailored to Chinese characteristics.

With the continuous improvement of urban governance systems and standards, the primary task of urban ecological governance has gradually shifted from reducing environmental pollution to improving the efficiency of green development. Therefore, improving the efficiency of urban green development has become a widely accepted consensus within the international community. In this context, the theory and empirical research of green development, which focuses on an intensive and efficient development model, have gradually attracted wide attention from various sectors [6, 7]. In 1987, the United Nations World Commission on Environment and Development first mentioned the concept of sustainable development in its report “Our Common Future” [8]. In 2005, at the fifth Asia-Pacific Conference on Environment and Development, the United Nations Economic and Social Commission for Asia and the Pacific proposed the concept of "green growth," defining it as "environmentally sustainable economic growth." In 2007, the United Nations Environment Program first defined a “green economy” as "an economy that values people and nature and creates decent, well-paying jobs.” With the international community’s growing deepening understanding of the interplay between economic activities, resources, and the environment, particularly in the wake of the 2008 international financial crisis, various initiatives such as the green economy, green New Deal, green growth, green transformation, and green development have been successively introduced to address the actual requirements of development and sustainability. In 2011, the Organization for Economic Cooperation and Development identified green development as the solution to economic growth and development, as well as the key to pre-venting environmental degradation, loss of biodiversity, and unsustainable use of natural resources [9]. In 2012, the United Nations Conference on Sustainable Development adopted “Devel-oping Green Economy” as tits theme, and explicitly guided the new development direction of “global economy to green transition.” As a crucial indicator of green development [10], green development efficiency comprehensively considers the resource consumption involved in regional social and economic development, which is conducive to objectively reflecting the real cost of regional sustainable development [11]. Research on green development efficiency has made considerable progress. Scholars have empirically analyzed the green development efficiency across various sectors, including finance, manufacturing, industry and agriculture [12–16], focusing on green economic efficiency, green production efficiency, green innovation efficiency, green technology efficiency and green water resource utilization efficiency [17–19]. In addition, researchers have employed panel Tobit regression models, Bootstrap truncated regression models, and spatial autoregressive models to explore the factors influencing factors of green development efficiency [20–22]. They found that economic development, industrial agglomeration, technological innovation, and urbanization had a remarkable impact on the improvement of urban green development efficiency [23–25].

In the context of green development, the “traditional manufacturing economy” has progressively shifted to the “service economy”, with the service industry becoming increasingly prominent in the national economy [26]. The producer services characterized by its knowledge intensity, energy efficiency, emission reduction, agglomeration and distribution, and a high degree of industrial integration, plays a crucial role in manufacturing and service industries, supporting the formation of modern service industry clusters that integrate upstream and downstream links such as R&D, production, storage, transportation and marketing [27]. Therefore, the agglomeration of producer services has gradually become a new driving force for the formation of the transformation of the industry from a production and manufacturing type to a production and service type, thereby enhancing green production efficiency and achieving high-quality growth [28]. With the evolution of economic development concepts and the international community’s emphasis on ecological protection, some studies have begun to focus on the impact of producer services on urban ecology, yielding several beneficial results. First, the desired outcomes of urban green development are influenced by the agglomeration of producer services. Several scholars have examined the effect of producer services on enhancing urban economic growth and enhancing economic performance. Their research indicates that producer services have emerged as a novel catalyst for promoting high-quality economic advancement [29, 30]. Second, the agglomeration of producer services plays a crucial role in minimizing the negative outputs of urban green development. Some researchers propose that producer services can effectively enhance the efficiency of urban green development and encourage the intensive and efficient utilization of resource elements [31, 32]. Third, the spatial spillover effects of producer services agglomeration contribute to the harmonious development of the urban economy and environment. Relevant studies indicate that advancements in modern communication technology have markedly reduced the impact of spatial distance on the location selection of producer service enterprises, enabling them to cluster effectively even over long distances [33]. Furthermore, some certain research highlights that producer services exhibit strong industrial integration and a noticeable trend toward agglomeration [34]. Producer services effectively enhance the external efficiency of the agglomeration process by leveraging technological innovations and other strategies during this process, thereby notably improving urban environmental benefits [35].

Overall, while previous research has theoretically and practically explored how producer services affect the efficiency of urban green development, the differences in impact and spatial effects among various types of producer services have not been thoroughly examined. This study addresses this gap by analyzing 284 cities above the prefecture level as a research unit. The influence of producer services agglomeration on urban eco-efficiency and its spatial effect is investigated from the perspective of agglomeration externality. The marginal contributions of this study include the following: (1) Establishing a theoretical framework for the impact of producer services agglomeration on green development efficiency, thereby enriching the theoretical extension of industry agglomeration research. (2) Utilizing the spatial panel Durbin model to examine the direct and indirect effects of producer services agglomeration on urban green development, which enriches the understanding of the externality differences in producer services agglomeration. (3) Incorporating the heterogeneity of geographical and economic locations into the analytical framework by decomposing producer services agglomeration by type, thereby identifying the detailed characteristics of the green development effects of producer services.

## Theoretical framework and research hypothesis

**Fig 1:**
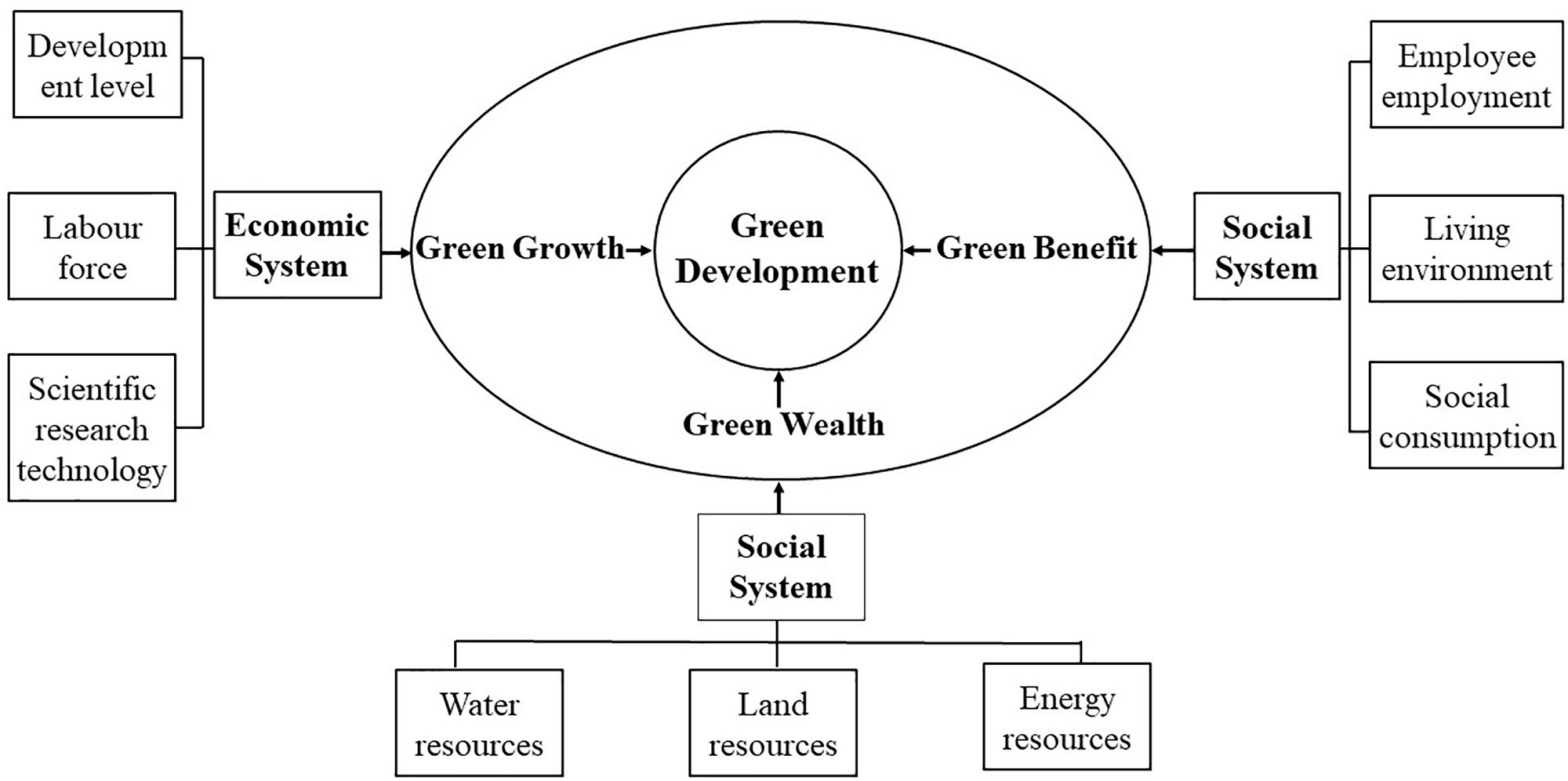
Theoretical framework of green development.

Green development has profound theoretical importance and practical direction in supporting and achieving global sustainable development goals [3]. Through literature review, the current definition of green development is vague and often emphasizes only one aspect, such as the economy, society, or nature [6, 36]. Therefore, this research aims to build on previous studies by integrating economic, social, and natural systems to redefine the theoretical framework of the concept of green development. The theoretical framework is structured into a three-circle interaction system comprising "a target layer, a connotation layer, and a reference layer" (Fig 1). The first layer is the base layer, comprising the economic, social, and natural systems. Within this circle, economic factors such as the level of economic development, labor force, and scientific research and technology create a new organization system that influences the operation of the economic system. In the social system, green benefits derived from employment, living environment, and social consumption form the social foundation for green development. The natural system includes water, land, and energy resources as its elements, forming the benchmark for green development within the natural circle. The second layer focuses on green welfare as the target, green wealth as the foundation, and green growth as the means, forming the connotation layer comprising "green growth-green welfare-green wealth". The third layer is the core layer, with the ultimate goal of achieving green development.

The interaction between the various circles supports the operation of the green development system. Green growth relies on green wealth, which generates economic and social benefits while continuously accumulating green wealth through the enhancement of green welfare. Green welfare serves as a developmental goal and a constraint. It originates from natural systems and forms the basis for the carrying capacity of the natural resource environment necessary for overall development. Green welfare has a carrying capacity boundary; exceeding this boundary can compromise the development of natural systems, undermining the material foundation for green wealth and green growth. The accumulation of green wealth is attributed to sustained green growth. The efficiency of urban green development directly impacts green wealth, green welfare, and green growth while also indirectly influencing economic, social, and natural systems. Therefore, green development is a sustainable development concept that encompasses green growth, green welfare, and green wealth, integrating the three major systems of the economy, society, and nature. This type of development aims to achieve a harmonious balance between economic growth, environmental protection, and social equity, emphasizing the reduction in negative environmental impacts, conservation of natural resources, improvement of resource efficiency, and promotion of social justice.

**Fig 2:**
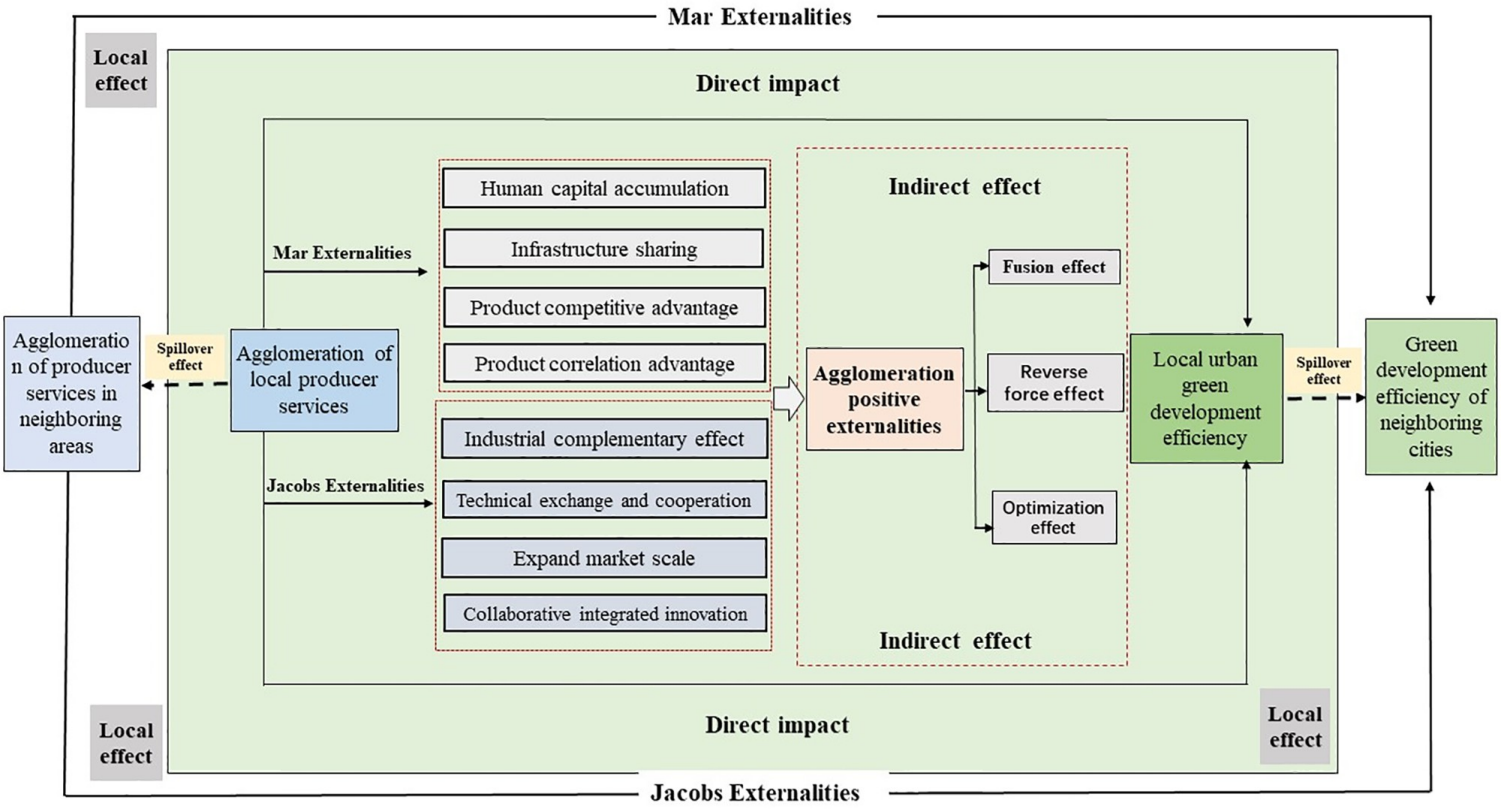
Theoretical framework of green development effects of producer services from the perspective of agglomeration.

Based on the interpretation and construction of the theoretical framework of green development, this paper establishes a theoretical model for analyzing the impact of producer services on green development from an agglomeration perspective (Fig 2). The agglomeration of industries is a notable geographical feature of economic activities that enhances production efficiency by stimulating external agglomeration effects [37]. As a representative modern service industry, the impact of producer service agglomeration on urban green development is mainly reflected in the external efficiency of agglomeration. Additionally, industrial agglomeration can be divided into specialized agglomeration and diversified agglomeration based on the method of clustering [38]. Research on specialized agglomeration dates back to Marshall, who identified labor market sharing, specialized inputs, and technology spillover as the primary sources of specialized agglomeration [39]. Scholars later supplemented the explanation of the externality of specialized agglomeration based on "increasing returns" in the endogenous economic growth model, forming the Mar externality theory of industrial agglomeration [40]. The specialized agglomeration of producer services enhances the positive externality efficiency of agglomeration, such as through professional capital accumulation, infrastructure sharing, and industrial association advantages. This strategy also strengthens the inter-industrial association effect by improving technological innovation capabilities and extending production links throughout the value chain [30]. Diversified agglomeration, or Jacobs externality, emphasizes that knowledge exchange and technological cooperation between different industries help enterprises expand their market scale and stimulate collaborative and integrated innovation [41]. The diversified agglomeration of producer services creates a circular causal effect through geographical proximity and relationship proximity by strengthening input-output links between different industries and complementarity in production factor matching. This causal effect acts as a "lubricant" connecting various industries, realizing industrial complementarity while expanding market scale through industrial division of labor and cooperation. Additionally, the spatial agglomeration of different industries creates a diversified environment, promoting technological exchanges, cooperation, and collaborative innovation between industries [29]. Therefore, the three effects of integration, inversion, and optimization caused by producer services agglomeration can indirectly affect the green development of local cities through industrial integration, industrial structure, development environment, external development and other pathways.

Furthermore, the agglomeration of producer services not only has direct and indirect effects on the effectiveness of green development in the local area but also creates an overflow impact on the green development of neighboring cities through technological and industrial linkages [42]. On the one hand, as a supportive industry for the development of service manufacturing and high-tech sectors, producer services directly influence local green technology efficiency. The three effects of integration, coercion, and optimization caused by the agglomeration of producer services can impact local green development efficiency through specific pathways, such as industrial integration, industrial structure, optimization of the development environment, and promotion of pollution control. On the other hand, producer services exert a positive spillover effect on the green development efficiency of neighboring cities through spatial, structural, and ecological effects associated with their agglomeration. Based on the above analysis, this paper proposes the following three hypotheses:

H1: Producer services agglomeration positively impacts urban green development efficiency and exhibits spatial spillover effects.
H2: The influence of specialized and diversified agglomeration of producer services on urban green development efficiency differs.
H3: The influence of producer services agglomeration on urban green development efficiency varies across industries and region.

## Data sources and research methods

### The collection and manipulation of data

The research data sources include two components: (1) Fundamental geographical data. The vector administrative boundary map is obtained from the National Basic Geographic Information Center’s China basic geographical information data (http://www.ngcc.cn/). Excluding Taiwan, Hong Kong, and Macao, this study focuses on 284 cities in China that are at or above the prefecture level. To ensure spatial consistency and facilitate analysis, certain cities with inconsistent time series data are excluded. (2) Socio-economic data. The agglomeration index for producer services’ specialization and diversification is determined by the number of employees in subsectors, as reported in the China City Statistical Yearbook (https://data.stats.gov.cn), and is computed using appropriate models. Data on fixed inventory, employee count in units, mean salary of urban workers, and urban green area primarily originate from the China City Statistical Yearbook (https://data.stats.gov.cn) and China Economic Net statistical database (https://db.cei.cn), spanning from 2002 to 2018. Some missing data are adjusted and supplemented by data from regional official websites. Furthermore, based on the Statistical Classification of Producer Services (2019) provided by the National Bureau of Statistics (https://www.stats.gov.cn) and in conjunction with previous studies [43], six categories of industries, including computer and software, finance, scientific research and technology, leasing and commerce services, transportation and postal services, and wholesale and retail trade, are taken as components of producer services.

### Research methods

#### Spatial agglomeration measurement model

Based on existing research, this paper divides the agglomeration mode of producer services into two types: specialized agglomeration and diversified agglomeration, and constructs a measurement model [44].

$$RZI_{j}=\sum_{s}\left.\left|\frac{E_{j,s}}{E_{j}}-\frac{E_{s}^{\prime }}{E^{\prime }}\right.\right|$$ (1)

Where: *E*_*j*,*s*_ indicates the employment number of producer services in city *j*; *E*_*j*_ indicates the total number of employments in city *j*; *E*^*’*^_*s*_ indicates the number of employment in producer services in other cities of the country except city *j*; *E*^*’*^ indicates the number of people employed in cities other than City *j* in the region.

Similarly, the diversification agglomeration measurement model is constructed, and its formula is as follows:

RDIj=∑sEj,sEj1∑s′=1,s′≠snEj,s′(Ej−Ej,s)21∑s′=1,s′≠snEs′(E−Es)2 (2)

Where: *E*_*j*,*s’*_ indicates the number of employments in other in other types of producer services in city *j* except for producer services s; *E*_*s*_ indicates the total number of people employed in producer services *s* nationwide; *E*_*j*_ indicates the total number of people employed in City *j*.

#### SBM-Undesirable model

During urbanization, besides the anticipated outcomes, certain unfavorable consequences like ecological contamination will also occur. The conventional radial DEA approach does not consider the slack variable component of the invalid DMU, leading to a bias in the efficiency measurement involving undesired output. To rectify the issues with Slack variables, Tone suggested the SBM-Undesirable Model as a solution. This model effectively addresses the problems related to slack and undesirable output of input-output variables, providing an accurate evaluation of China’s urban green development efficiency [45]. The formula is as follows:

ρ=min1−1N∑n=1Nsnxxk′nt′1+1M+1∑m=1Msmyyk′mt′+∑i=1Isibbk′it′s.t.∑t=1T∑k=1Kzktxknt+snx=xk′nt′,(n=1,2⋯,N)∑t=1T∑k=1Kzktxkmt+smy=yk′mt′,(m=1,2⋯,M)∑t=1T∑k=1Kzktbkit+sib=bk′it′,(i=1,2⋯,N)zkt≥0,snx≥0,smy≥0,sib≥0,(k=1,2⋯,K) (3)

Where: The target efficiency is denoted by *ρ*, while *N*, *M*, and *I* correspond to the number of inputs, expected output, and unexpected output respectively. (*x*^*i*^_*k ‘n*_, *y*^*i*^_*k ‘*_*n*, *b*^*i*^_*k ‘n*_) denotes the value of *k* ‘decision unit’s input-output in the *t* ‘period; (*S*^*x*^_*n*_, *S*^*y*^_*n*_, *S*^*b*^_*n*_) refer to the relaxation of input, anticipated output, and unforeseen output, correspondingly.

#### Evaluation index system of urban green development

Based on the "three-circle system theory" of green development mentioned above, the input-output model is used to establish an index system for measuring the efficiency of urban green development in China from both input and output perspectives (Table 1). In terms of input, scholars mostly measure it from the perspectives of labor, capital, and technology [2, 45]. Considering the significant role of natural factors in urban green development, this article incorporates elements such as water, land, and energy, along with socio-economic factors like capital, labor, technology, and resources, into the calculation of factor inputs. Specifically, capital input is represented by capital stock, labor input by the total number of employees over the years, technical input by the financial expenditure on science, technology, and education in various regions, and the resource input by the total consumption of water, soil, and energy. In terms of expected output, existing studies mostly measure economic output and pay less attention to the social effects and ecological benefits brought by green development [3, 46]. Therefore, by comprehensively considering the economy, society, and ecology output benefits, this paper selects the GDP of each region to reflect economic benefits, a social benefit index to reflects social output, and an environmental benefit index to reflect environmental output. Additionally, considering the value loss caused by waste discharge to the ecological environment during the development process, the environmental pollution index is included in the evaluation system as the non-expected output factor, with resource and environmental constraints taken as important factors in evaluating the efficiency of green development.

**Table 1:** Evaluation system of urban green development efficiency in China.

Type	Primary index	Secondary index	Three-level index
Input indicator	Capital element	Stock of fixed capital	Total fixed assets of society
	Factors of labor force	Number of employees	Number of units employed at the end of the year
	Technical element	Expenditure on science and technology	Total expenditure on science and technology at the end of the year
	Resource element	Total water, land and energy consumption	Total water supply. Urban built-up area. Electricity consumption of the whole society. Artificial and natural gas supply. Liquefied gas supply
Output indicator	Expected output	Economic benefit	Gross domestic product
		Social benefit	Average wages of urban workers. Total retail sales of consumer goods
		Environmental benefit	Urban green area. Green coverage rate. Comprehensive utilization rate of industrial solid waste. Centralized treatment rate if sewage treatment plants. Harmless treatment rate of household garbage
	Non-expected output	Environmental pollution	Industrial wastewater discharge. Industrial SO_2_ discharge. Industrial soot discharge

#### Spatial Durbin model and variable selection

Considering that the external efficiency of industrial agglomeration not only affects the development of the local area, but may also have spillover effects on the development of neighboring areas [30]. This study uses the spatial Durbin model to deconstruct the externality efficiency of industrial agglomeration from two perspectives: the direct effect of industrial agglomeration on the local area and the indirect effect on surrounding regions.

$$lnUGDE_{it}=\alpha_{i}+\rho \sum_{i=1}^{n}W_{ij}\;ln\;UGDE_{it}+\varphi X_{it}+\theta \sum_{i=1}^{n}W_{ij}X_{it}+\mu_{i}+\delta_{i}+\varepsilon_{it}$$ (4)

Where: *UGDE*_*it*_ represents the green development efficiency of city *i* in year *t*; *α*_*i*_ is a constant term; *ρ* is the spatial regression coefficient; *θ* represents the regression coefficient of the independent variable; *X* is the independent variable; *W*_*ij*_ *X*_*it*_ is the coefficient of its spatial lag term. *μ*_*i*_ and *δ*_*i*_ are city fixed effect and time fixed effect, respectively.*ε*_*it*_ is a random disturbance term; *W*_*ij*_ represents the spatial weight matrix.

Taking urban green development efficiency (UGDE) as the explanatory variable, the specialized agglomeration (Mar) and diversified agglomeration (Jac) of the producer services are selected as the core explanatory variables (Table 2). In addition, based on the existing literature, this paper controls for a series of other natural background factors and humanistic and social factors that may affect the efficiency of urban green development. In terms of natural background factors, annual precipitation and PM2.5 concentration are selected to represent urban precipitation conditions (PRE) and environmental quality (EQ). In terms of humanistic and social factors, per capita GDP (RGDP) is used to measure macroeconomic strength, the proportion of scientific and technological expenditure in regional fiscal revenue is used to reflect technological innovation (TE), and the number of college students per ten thousand people is used to measure human capital level (Hum).

**Table 2:** Main variables of the model.

Variable type	Variable	Symbol	Variable declaration
Explained variable	Urban green development efficiency	*UGDE*	
Core explanatory variable	Mar externalities	*Mar*	Agglomeration of specialization in producer services
	Jacobs externality	*Jac*	Diversified agglomeration of producer services
Control variable	Urban precipitation	*Pre*	Annual precipitation
	Environmental quality	*EQ*	PM2.5 concentration
	Macroeconomic strength	*RGDP*	Per capita GDP
	Technological innovation	*TE*	Science and technology expenditure as a proportion of local government revenue
	Human capital level	*Hum*	Number of college students per 10,000

## Spatiotemporal evolution characteristics of producer services agglomeration and urban green development efficiency in China

In the research process, three time points of 2002, 2010, and 2018 were selected to describe the spatial pattern evolution progress of China’s producer services agglomeration and urban green development efficiency. This study adopts the partition thresholds from the 2002 measurement results as benchmarks to consistently classify the agglomeration and efficiency grades for 2010 and 2018 and ensure comparability of measurement results across different years in the long-term spatial pattern evolution analysis.

### Spatiotemporal evolution characteristics of agglomeration of producer services

Industrial agglomeration is considered an effective spatial organization model that mitigates distance constraints between enterprises through external economic benefits [45]. This research employs spatial visualization methods to illustrate and analyze the specialization and diversification characteristics of producer services agglomeration in different Chinese cities across different years.

#### Spatiotemporal evolution characteristics of specialized agglomeration

**Fig 3:**
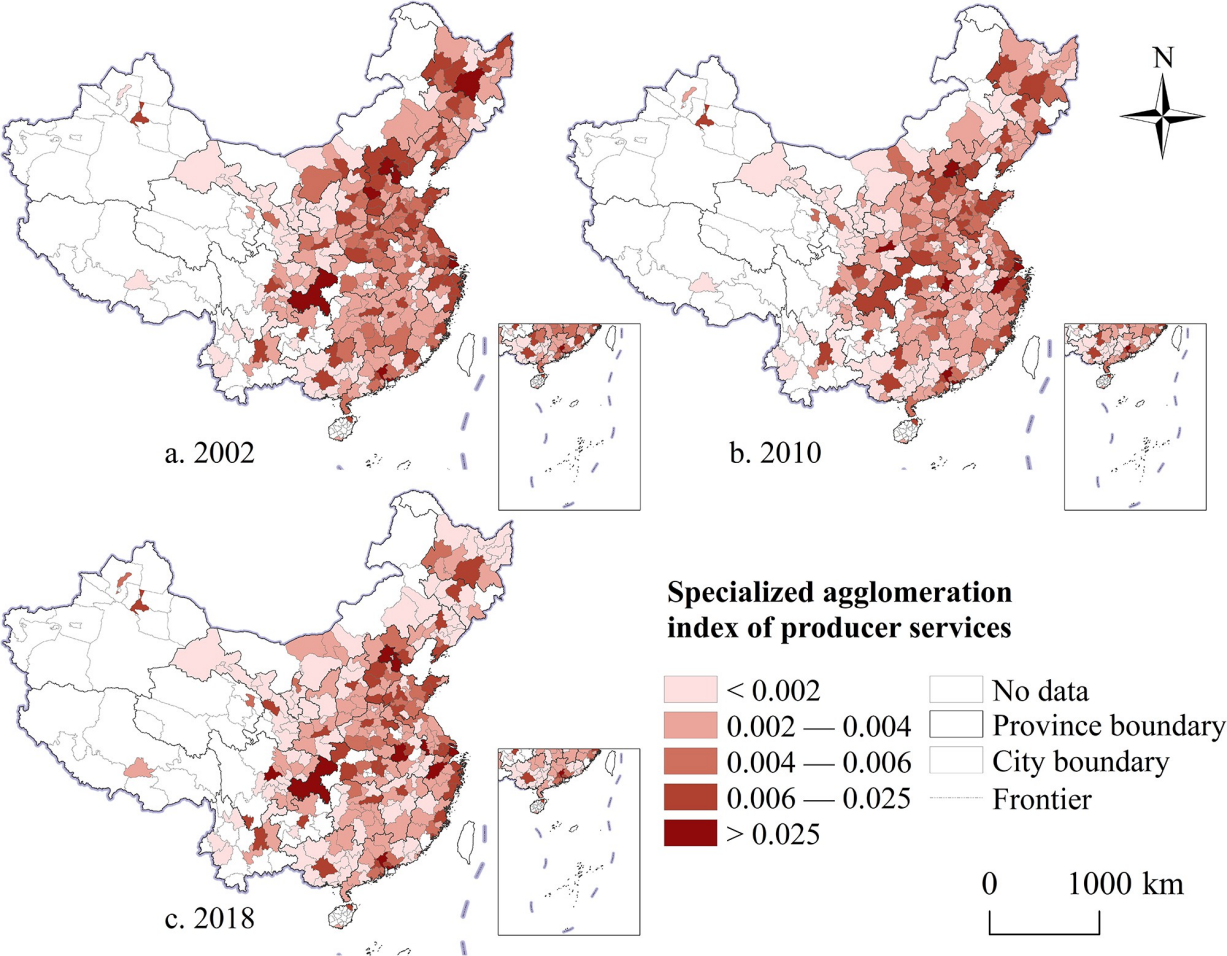
The spatial and temporal evolution trend of the specialized agglomeration of producer services in China from 2002 to 2018. Note: Map created by the authors using open-access base data from the National Geoinformation Public Service Platform ’Tianditu’ (http://example-url). Data are non-confidential and shared under relevant national policies (Notice on the Issuance of the Management Measures for the Provision and Use of Non-Confidential Surveying and Mapping Geographic Information Results, Article 20).

Measurement results reveal that from 2002 to 2018, the level of professional agglomeration of China’s producer services exhibited a downward trend, with a logarithmic decline coefficient of 0.034. The study employed the natural discontinuity method to visually analyze the agglomeration levels of producer services at the city scale and further assess the spatial disparities of producer services agglomeration in China. The analysis indicates that, although the overall specialization pattern of producer services in China during the study period exhibited characteristics of "east higher than west and north higher than south" variations across different periods emerged. In 2002, cities with a high level of professional agglomeration of producer services were mainly distributed in urban agglomerations located in the eastern coastal areas (Fig 3a). During this period, various producer service activities were primarily provided by manufacturing enterprises, with the growth of producer services heavily dependent on the demand for production means from these enterprises. Consequently, cities with a stronger industrial base created favorable conditions for the specialization of producer services. In 2010, although the level of specialized agglomeration in China’s producer services showed a slight decline compared to the previous period, the overall pattern remained stable (Fig 3b). As the role of producer services in urban development became more prominent, the spatial disparities in their professional concentration levels gradually narrowed, indicating a shift from "unbalanced" to "balanced" development. By 2018, most cities exhibited varying degrees of decline in professional agglomeration (Fig 3c). This trend may be related to policies aimed at expediting the removal of outdated production capacities and facilitating a shift in economic development models.

**Fig 4:**
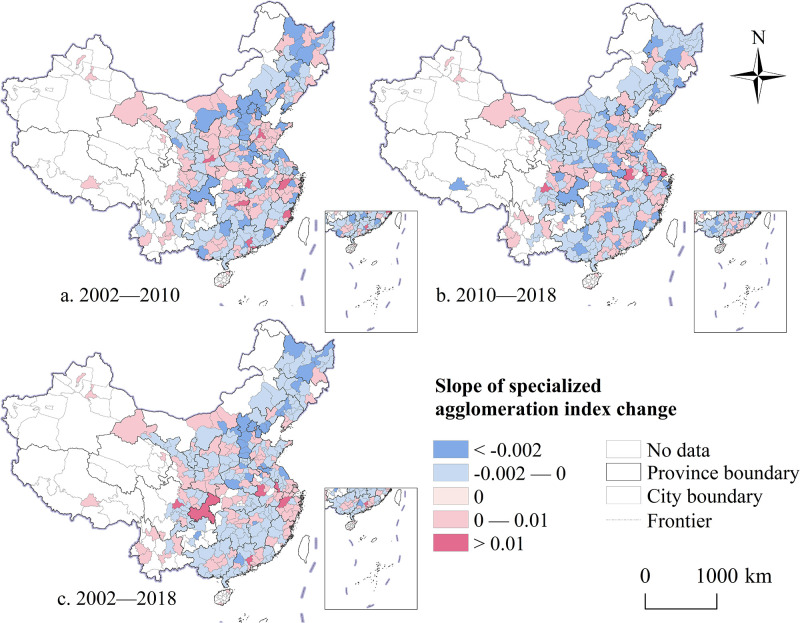
The spatio-temporal comparison of the specialized agglomeration level of China’s producer services from 2002 to 2018. Note: Map created by the authors using open-access base data from the National Geoinformation Public Service Platform ’Tianditu’ (http://example-url). Data are non-confidential and shared under relevant national policies (Notice on the Issuance of the Management Measures for the Provision and Use of Non-Confidential Surveying and Mapping Geographic Information Results, Article 20).

Analysis reveals that cities with an increased level of producer services specialization from 2002 to 2010 were primarily located in the core areas of urban agglomerations or metropolitan areas (Fig 4a). Furthermore, as China’s economic development underwent a “soft landing,” some cities previously dominated by heavy industry gradually shifted to diversified industrial types, leading to a reduction in the specialization level of producer services as intermediate inputs. From 2010 to 2018, the number of cities with improved specialization levels increased, with these cities mainly located on the periphery of urban agglomerations (Fig 4b). The distribution pattern of cities experiencing more noticeable declines in specialization agglomeration became more dispersed, with many being cities with higher levels of economic development. Overall, during the study period, changes in the specialization level of producer services exhibited a dynamic development trend and certain regional regularity (Fig 4c).

#### Spatiotemporal evolution of diversified agglomeration

**Fig 5:**
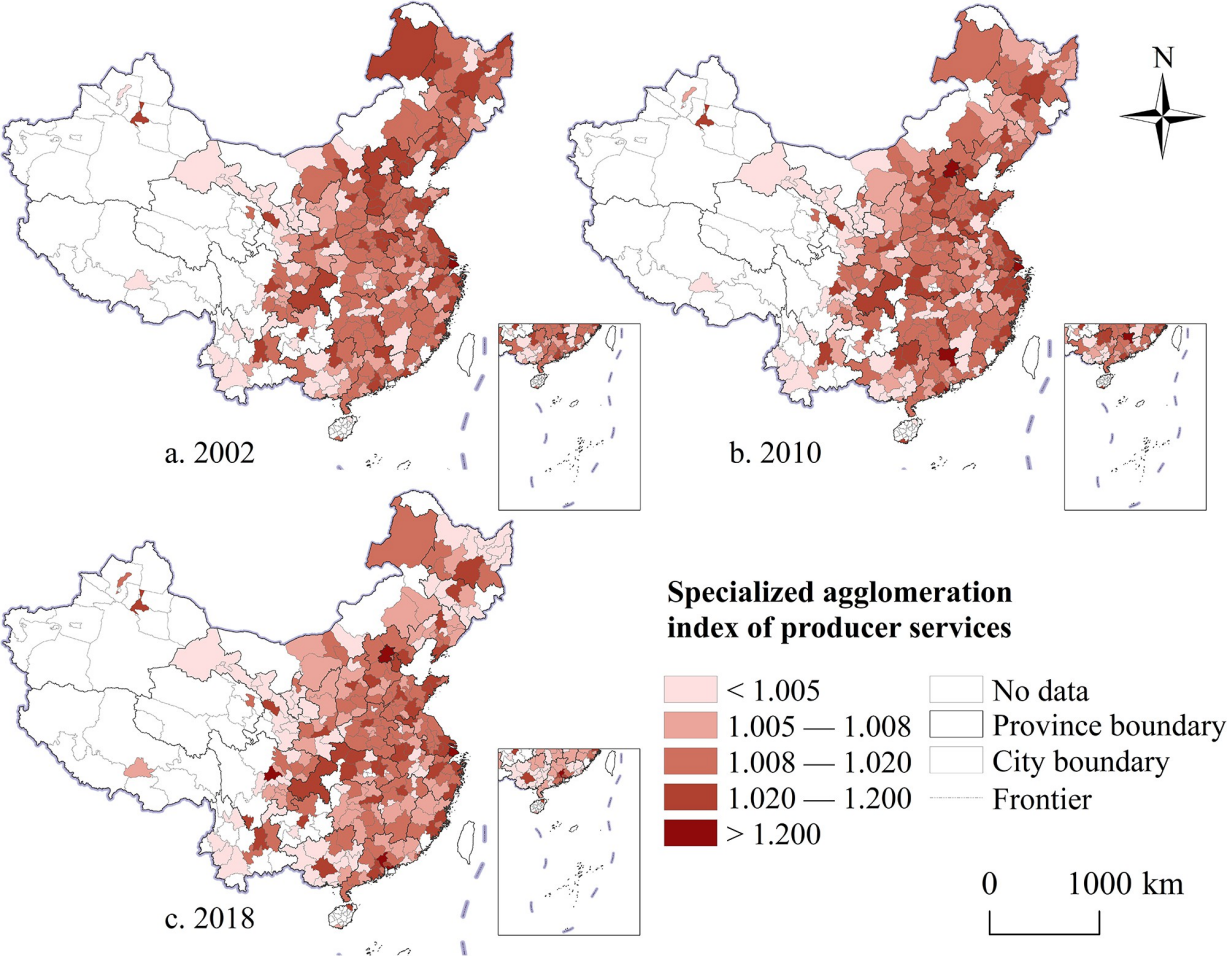
Spatiotemporal evolution trend of diversified agglomeration of Chinese urban producer services from 2002 to 2018. Note: Map created by the authors using open-access base data from the National Geoinformation Public Service Platform ’Tianditu’ (http://example-url). Data are non-confidential and shared under relevant national policies (Notice on the Issuance of the Management Measures for the Provision and Use of Non-Confidential Surveying and Mapping Geographic Information Results, Article 20).

The overall trend of diversified agglomeration in Chinese urban producer services has consistently increased throughout the research period, with a logarithmic rise coefficient of 0.008. From 2002 to 2018, the diversified agglomeration of producer services in China exhibited spatial characteristics of being “dispersed over an extensive area with localized concentrations.” In 2002, the diversified agglomeration of Chinese producer services displayed notable regional heterogeneity (Fig 5a). Regions with remarkably diversified agglomeration were primarily distributed in the eastern coastal areas and the core zones of urban agglomerations. These cities, benefiting from favorable locations and policies, had higher priorities for advancing technology, attracting foreign investments, and upgrading industries. Additionally, their robust industrial structure created an environment conducive to the diverse concentration of producer services. By 2010, the overall level of diversified agglomeration in China’s producer services had notably improved (Fig 5b). With the continuous optimization of China’s industrial structure, large cities with higher economic development levels drove the diversified growth of related producer services through their strong manufacturing base. Furthermore, the expansion and spillover of productive service functions from central cities promoted the continuous growth and diversification of producer services in several smaller cities. Compared with the previous period, most economically developed cities showed an obvious downward trend, and the balance of diversified agglomeration levels between regions gradually increased (Fig 5c). As the industrial structure of coastal cities evolved, traditional manufacturing industries began migrating from the eastern coast to the inland northwest areas. Consequently, as industrialization accelerated in these inland areas, their manufacturing systems became more comprehensive, and producer services associated with them increasingly exhibited a trend toward diversified agglomeration.

**Fig 6:**
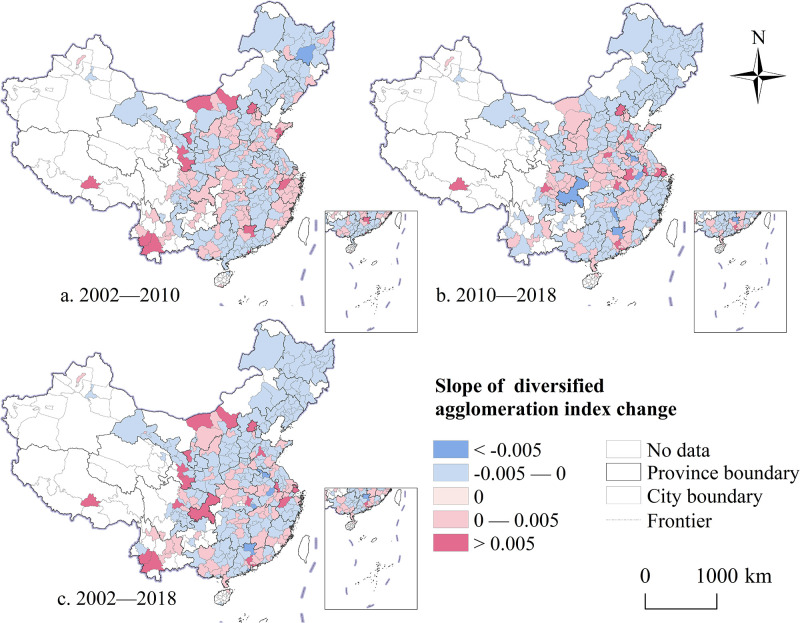
The spatio-temporal comparison of diversified agglomeration level of producer services in Chinese cities from 2002 to 2018. Note: Map created by the authors using open-access base data from the National Geoinformation Public Service Platform ’Tianditu’ (http://example-url). Data are non-confidential and shared under relevant national policies (Notice on the Issuance of the Management Measures for the Provision and Use of Non-Confidential Surveying and Mapping Geographic Information Results, Article 20).

From the perspective of the spatiotemporal changes in the diversified agglomeration level of Chinese cities, 124 cities experienced an increase in diversified agglomeration from 2002 to 2010, mainly distributed in the Hetao Plain, Shandong Peninsula urban agglomeration, Chengdu—Chongqing metropolitan area, and several cities along the southeast coast (Fig 6a). From 2010 to 2018, compared to the previous period, cities with increasing levels of diversified agglomeration gradually shifted from the eastern coastal areas to the central and western regions (Fig 6b). Overall, the diversified agglomeration level of producer services dynamically improved during the research period. Specifically, 106 cities exhibited an increase in diversified agglomeration, while the overall pattern remained largely stable despite a slight decline in the remaining cities (Fig 6c). This measurement indicates that while diversified agglomeration of producer services facilitates knowledge sharing and cross-border exchanges between industries, it is influenced by factors such as industrial structure, location conditions, and development mode, leading to considerable differences in diversified agglomeration levels between cities. Moreover, the levels of diversified agglomeration in different cities exhibited growth and decline trends.

### Spatiotemporal evolution characteristics of urban green development efficiency

**Fig 7:**
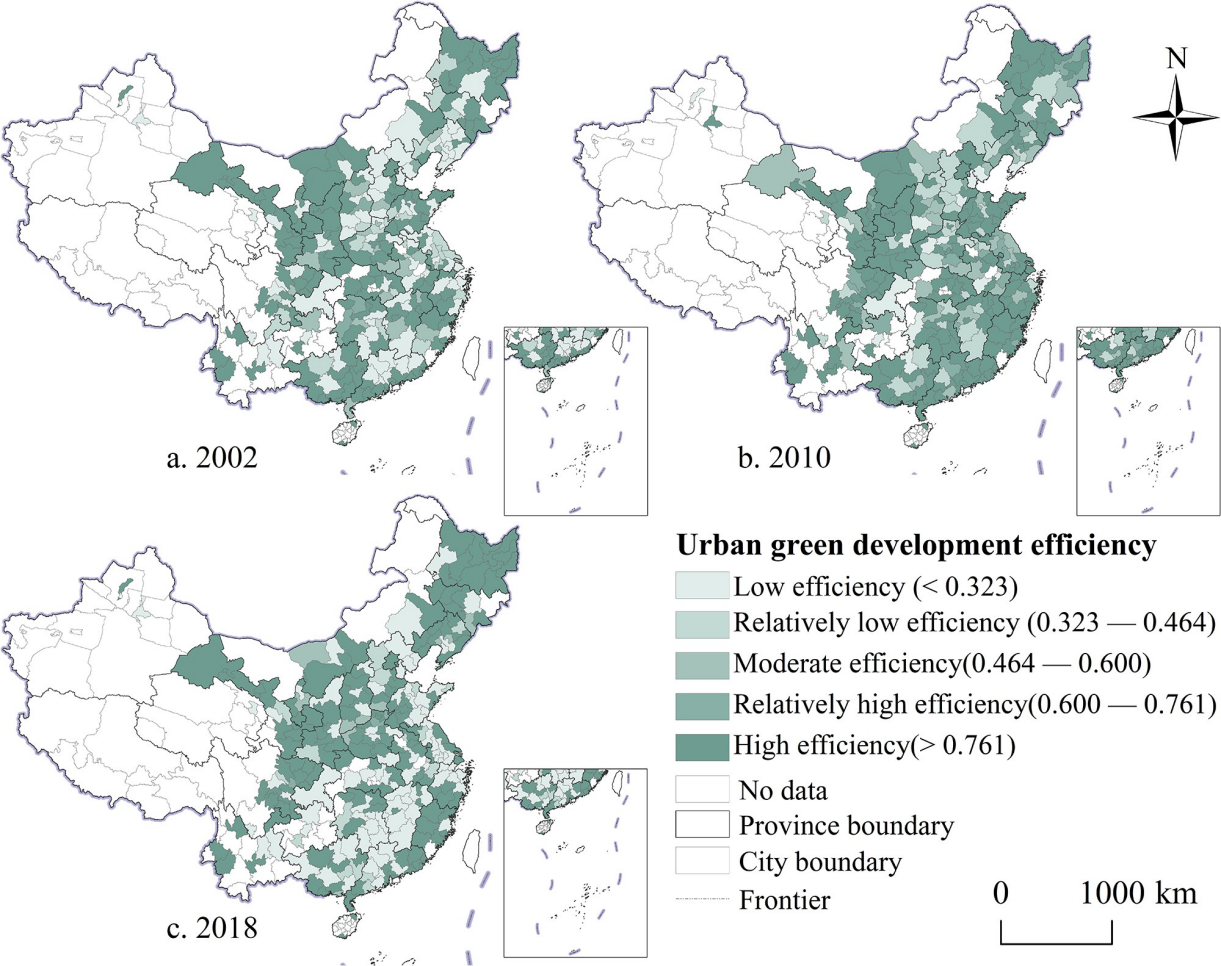
The spatio-temporal evolution trend of the UGDE of Chinese cities from 2002 to 2018. Note: Map created by the authors using open-access base data from the National Geoinformation Public Service Platform ’Tianditu’ (http://example-url). Data are non-confidential and shared under relevant national policies (Notice on the Issuance of the Management Measures for the Provision and Use of Non-Confidential Surveying and Mapping Geographic Information Results, Article 20).

The efficiency of urban green development in China exhibited a dynamic upward trend, with a logarithmic increase coefficient of 0.030. According to the calculation results, the green development efficiency was categorized into five levels: low efficiency (<0.323), relatively low efficiency (0.323–0.464), moderate efficiency (0.464–0.600), relatively high efficiency (0.600–0.761), and high efficiency (>0.761). Analysis of measurements reveals distinct regional differences and dynamic development characteristics. At three time points—2002, 2010 and 2018—high efficiency cities were primarily located in the eastern coastal regions, the upper and middle reaches of the Yellow River, the middle reaches of the Yangtze River, and the northeast region (Fig 7). Benefiting from favorable location and policy advantages, these high-efficiency cities have achieved remarkable advancements in industrial structure and environmental governance. Throughout this process, the industrial structure of these cities has largely shifted from labor-intensive to capital-intensive and knowledge-technology-intensive industries. This shift has increasingly emphasized the reliance on technology and capital, thereby mitigating constraints related to resources, environment, and labor costs. Additionally, the allocation of input factors has been transformed and upgraded, leading to increased expected outputs, and a reduction in non-expected outputs, such as environmental pollution. Therefore, these cities have maintained a high level of urban green development efficiency. Relatively high efficiency cities are distributed around high-efficiency areas, forming several concentrated zones, such as the periphery of the Yangtze River Delta urban agglomeration, the Shandong Peninsula urban agglomeration, and the Jianghuai urban agglomeration. Medium-efficiency cities are widely distributed in most parts of China, but their distribution range has gradually decreased, with notable improvements in the middle reaches of the Yangtze River. Provinces such as Henan, Shaanxi, Sichuan, and Hebei have maintained a medium efficiency status with minimal changes during the study period. Low efficiency cities are mainly distributed in some cities in Yunnan, Guizhou, and Guangxi.

**Fig 8:**
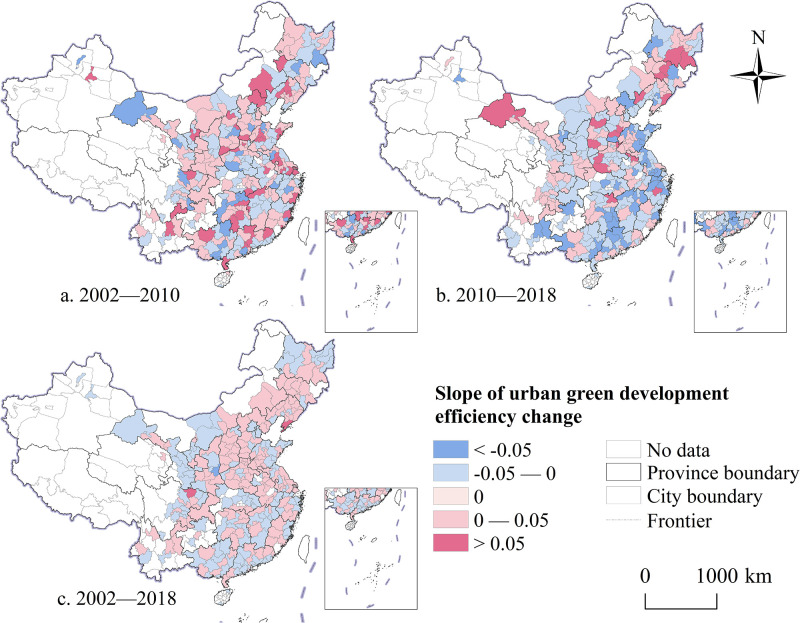
Spatio-temporal comparison of the green development efficiency of Chinese cities from 2002 to 2018. Note: Map created by the authors using open-access base data from the National Geoinformation Public Service Platform ’Tianditu’ (http://example-url). Data are non-confidential and shared under relevant national policies (Notice on the Issuance of the Management Measures for the Provision and Use of Non-Confidential Surveying and Mapping Geographic Information Results, Article 20).

Despite notable regional differences in the changes in green development efficiency across cities over each time period, a certain spatial correlation is evident. From 2002 to 2010, the green development efficiency of 156 cities improved to varying extents, primarily in cities within Liaoning, Hebei, Shaanxi, Sichuan, Fujian, Guangdong, Jiangsu, and Shandong provinces. During this period, 124 cities experienced varying degrees of decline in green development efficiency, mostly located on the periphery of urban agglomerations or metropolitan areas (Fig 8a). Except for cities such as Hengshui, Huaihua, Mudanjiang, Putian, and Jiuquan, which had relatively low green development efficiency, most cities saw slight decreases but remained relatively stable. From 2010 to 2018, the number of cities with increasing green development efficiency decreased compared with the previous period (Fig 8b). Overall, except for a small number of cities with a relatively noticeable decline in green development efficiency, other cities maintained stability and exhibited an overall trend of dynamic growth during the study period (Fig 8c). This finding indicates that improvements in green development efficiency drive cities to transform their economic development models and pursue green development. Consequently, the advancement of a green economy is increasingly becoming a prevalent trend in future urban development.

## The influence of producer services agglomeration on urban green development efficiency

### Spatial effects based on the whole sample

In addition to using the spatial Durbin model (SDM) to measure the impacts of producer services agglomeration on urban green development efficiency, the ordinary panel fixed effect model is also used as a reference for testing to examine the validity of the measurement results (Table 3).

**Table 3:** The influence of producer services agglomeration on UGDE and its spatial spillover effects: Full sample.

Effect type	Variable	SDM		FE	
		(1)	(2)	(3)	(4)
Direct effect	lnMar	0.032***		0.028***	
		7.507		5.032	
	lnJac		0.036***		0.030**
			8.309		2.417
	lnPre	0.029**	0.012*	0.034**	0.018*
		2.305	1.302	2.906	1.105
	lnEQ	-0.015***	-0.106***	-0.039	-0.036**
		-4.503	-9.215	-0.503	-2.572
	lnRGDP	0.017**	0.028*	0.032*	0.045**
		2.106	1.308	1.529	2.806
	lnTE	0.025**	0.015**	0.023*	0.028**
		2.302	2.028	1.905	2.076
	lnHum	0.031**	0.023**	0.045*	0.013**
		2.558	1.605	1.703	2.098
Indirect effect	lnMar	0.046***			
		9.853			
	lnJac		0.026***		
			6.392		
	lnPre	0.018*	0.016**		
		1.206	2.105		
	lnEQ	-0.058**	-0.030***		
		-2.832	-7.305		
	lnRGDP	0.023**	0.031**		
		2.179	2.780		
	lnTE	0.036***	0.027***		
		8.205	6.593		
	lnHum	0.029**	0.038**		
		2.180	2.509		

Note:

* means p < 0.10,

** means p < 0.05,

*** means p < 0.01.

From the perspective of direct effects, the measurement results of both models indicate that the diversified and specialized agglomeration of producer services have varying degrees of influence on the improvement of urban green development efficiency. According to the results of the SDM, a 1% increase in the level of diversified and specialized agglomeration of producer services leads to a 3.2% and 2.9% increase in urban green development efficiency, respectively. This result indicates that the agglomeration of producer services has a notable positive impact on enhancing urban green development efficiency. Specialized agglomeration promotes production specialization and competitive advantages within related industries, thereby advancing urban green development. Conversely, diversified agglomeration creates a varied production and service environment for regional industrial development, which minimizes production costs, enhances regional production efficiency, and supports industrial upgrading. A comparison shows that specialized agglomeration has a greater impact on urban green development efficiency than diversified agglomeration. This phenomenon may be because, despite the rapid expansion in the number of producer services, their development quality and integration level with related industries still need improvement. This limitation hinders the full potential of diversified agglomeration in boosting urban green development efficiency. Furthermore, specialization agglomeration enhances the innovation capability and core competitiveness of the industry, positively influencing the research, development, and application of new technologies. The widespread adoption of these new technologies improves production efficiency and reduces resource consumption, ultimately promoting new quality productivity and facilitating the transition from old to new growth drivers.

From the perspective of indirect effects, specialized agglomeration in producer services exhibits a notably positive spatial spillover effect on the efficiency of urban green development, with a coefficient value greater than corresponding direct effects. This result indicates that the flow of resources within the region enhances industrial collaboration between cities, creating a virtuous cycle in the agglomeration environment. Measurement results indicate that a 1% increase in the level of professional agglomeration of producer services indirectly leads to a 4.6% increase in the efficiency of urban green development. The measurement result shows that within a virtuous circle of agglomeration, the flow of resources within the region promotes industrial cooperation among cities, which enhances resource supply and improves urban green development efficiency. In contrast, while diversified agglomeration of producer services also positively contributes to the green development efficiency of neighboring cities, its impact is comparatively lower than that of specialized agglomeration. Specifically, a 1% increase in the level of diversified agglomeration of producer services leads to a 2.6% increase in the green development efficiency of surrounding cities. This result indicates that while diversified agglomeration currently contributes to the improvement of urban green development efficiency in neighboring cities through inter-city connections, further enhancement is still necessary.

### Decomposition of spatial effects based on type differences

Considering the distinct industrial characteristics of each subcategory within producer services, different types of industrial agglomeration may impact urban green development efficiency in various ways. Based on existing research [43], producer services are categorized into three types: advanced producer services, modern producer services, and traditional producer services. This study empirically analyzes the influence of various types of producer services on the effectiveness of urban green development (Table 4).

**Table 4:** The influence of producer services agglomeration on the UGDE and spatial spillover effects: Differences by type.

Effect type	Variable	Traditional type		Modern type		Advanced type	
		(1)	(2)	(1)	(2)	(1)	(2)
Direct effect	lnMar	0.035***		0.042***		0.059*	
		6.373		8.106		1.607	
	lnJac		0.023*		0.035**		0.018**
			1.309		2.497		2.174
	lnPre	0.012**	0.017**	0.102*	0.015**	0.035**	0.049***
		2.108	2.259	1.805	2.207	2.950	5.603
	lnEQ	-0.026***	0.025**	-0.016*	-0.062***	-0.037**	-0.042***
		-4.509	2.628	-1.029	-9.017	-2.194	-7.953
	lnRGDP	0.038**	0.014*	0.042	0.020**	0.042**	0.018***
		2.230	1.207	1.025	2.035	2.605	3.605
	lnTE	-0.019*	0.037**	0.029***	0.016*	0.023**	0.055***
		-1.303	2.162	5.179	1.330	2.104	8.580
	lnHum	0.026*	0.018**	0.016**	0.020*	0.022	0.037***
		1.306	2.320	2.516	1.525	0.805	6.553
Indirect effect	lnMar	0.030*		0.027***		0.016***	
		1.406		5.036		6.903	
	lnJac		0.019**		0.015*	0.132	0.031***
			2.021		1.303	0.958	6.219
	lnPre	0.016*	0.031	0.019*	0.031	0.025*	0.049**
		1.294	0.640	1.804	0.826	1.384	2.593
	lnEQ	-0.017*	-0.018*	-0.027**	0.015	0.033	-0.152*
		-1.026	-1.205	-2.605	0.928	0.293	-0.263
	lnRGDP	0.035**	0.130**	0.027***	0.042***	0.040**	0.108***
		1.296	2.309	5.029	3.608	2.094	7.306
	lnTE	0.017***	0.026**	0.031**	0.011*	0.025*	0.043**
		3.852	2.908	2.593	1.253	1.908	1.552
	lnHum	0.028**	0.017**	0.039*	0.042*	0.036**	0.072**
		2.508	2.858	1.307	1.793	2.506	3.158

Note:

* means p < 0.10,

** means p < 0.05,

*** means p < 0.01.

Measurement results reveal that the specialization of traditional producer services notably enhances urban green development efficiency. Specifically, a 1% increase in the specialization level of these cities results in a 3.5% improvement in urban green development efficiency. This measurement result indicates that the specialization of traditional producer services promotes the agglomeration of regional production resources and improves production efficiency. In contrast, while traditional producer services effectively improve local environment performance, their impact on neighboring cities through spatial spillover is limited. Furthermore, the effects of diversified agglomeration in traditional producer services are not substantial. This finding may be due to the labor-intensive nature of traditional producer services often lacking strong innovation capabilities and technical sophistication. Consequently, their attractiveness to neighboring areas is relatively weak, leading to a narrow service scope. Therefore, diversified agglomeration may raise production and transaction costs, impeding the spatial spillover impact of these services.

The specialized agglomeration of modern producer services positively impacts direct and indirect effects on urban green development efficiency. Specifically, a 1% increase in the specialization level of contemporary producer services leads to a 4.2% improvement in the efficiency of local urban green development and a 2.7% increase in neighboring cities. This effect is attributed to the crucial role of modern producer services in China’s urban economic system. Despite the transition of China’s economic progress model from high-speed growth to high-quality development, some industrial sectors still need substantial capital and support from business services due to the impact of the original industrial structure. In addition, with the promotion of the cross-regional layout of enterprise organizational structure and the division of labor and collaboration between regions, the professional agglomeration of modern producer services has also spread to neighboring areas through interregional exchanges, enhancing the eco-friendly development effectiveness of neighboring areas through spillover impacts. In contrast, the diversified agglomeration of modern producer services has a minimal effect on the green development efficiency of cities.

Although the specialized agglomeration of advanced producer services positively impacts urban green development efficiency, it only meets the 5% significance level in the model. This result indicates that the influence of specialized agglomeration of advanced producer services on the green efficiency of local cities is relatively modest. The external effects of this specialization are not fully realized due to factors such as agglomeration level and economic development. Regarding the diversified agglomeration of advanced producer services, despite its slight enhancement of green development efficiency of local cities, the overall effect is insufficient. In terms of indirect effects, advanced producer services play a crucial role in upgrading the industrial structure and production technology levels in surrounding areas through their strong radiative effects. Measurement results indicate that a 1% increase in the level of specialization and diversification of advanced producer services promotes the green development efficiency of neighboring cities by 1.6% and 3.1%, respectively. This result mainly stems from the development of the digital economy in this information age, which offers a broad market for the diversified agglomeration of producer services with intermediate input attributes, thereby promoting interregional urban green development efficiency improvement.

### Spatial effects of producer services agglomeration from the perspective of location

#### Spatial effects decomposition based on the differences in geographical location

Under the influence of topography, history, policy, and other factors, China has evolved into three major regions: the eastern coastal zone, the central zone, and the western zone. Considering these regional disparities, this study assesses the influence of producer services on the efficiency of urban green development across the three main regions (Table 5).

**Table 5:** The influence of producer services agglomeration on UGDE efficiency and spatial spillover effects: Regional differences.

Effect type	Variable	Eastern region		Central region		Western region	
		(1)	(2)	(1)	(2)	(1)	(2)
Direct effect	lnMar	0.062***		0.027***		0.109***	
		7.508		5.092		9.623	
	lnJac		0.128***		0.033*		0.019**
			10.207		1.936		2.028
	lnPre	0.026*	0.059	0.042*	0.017	0.021	0.033
		1.497	0.793	1.793	0.632	0.260	0.429
	lnEQ	-0.059**	-0.105*	-0.061**	-0.027*	-0.012	-0.018
		-2.903	-2.815	-2.377	-1.706	-0.193	-0.151
	lnRGDP	0.103*	0.041	0.127**	0.052*	0.019***	0.029***
		1.558	0.739	2.623	1.809	5.209	6.158
	lnTE	0.057***	0.072	0.035**	0.064*	0.031*	0.022
		6.437	0.876	2.383	1.980	1.306	0.309
	lnHum	0.037**	0.075	0.046*	0.022*	0.025	0.015*
		2.702	0.508	1.627	1.540	0.339	1.093
Indirect effect	lnMar	0.068***		0.062**		0.071**	
		8.629		2.303		2.605	
	lnJac		0.083***		0.049*		0.031**
			9.631		1.698		2.383
	lnPre	0.023	0.034*	0.058**	0.073*	0.024	0.028*
		0.286	1.742	3.804	1.972	0.335	1.507
	lnEQ	-0.049***	-0.072**	-0.037	-0.028*	0.025	0.017
		-7.635	-2.590	-0.597	-1.232	0.314	0.240
	lnRGDP	0.021***	0.107***	0.079**	0.103**	0.013*	0.034*
		9.139	5.902	2.036	2.405	1.025	1.558
	lnTE	0.045***	0.062***	0.058**	0.027*	0.012*	0.028
		5.709	4.094	2.715	1.013	1.052	0.305
	lnHum	0.032**	0.082*	0.041	0.039**	0.017	0.049*
		2.177	1.705	0.275	2.507	0.204	1.623

Note:

* means p < 0.10,

** means p < 0.05,

*** means p < 0.01.

The measurement results reveal that the impact of producer services agglomeration on urban green development efficiency varies across three economic major regions. Specifically, in eastern China, diversified and specialized agglomeration of producer services notably improve urban green development efficiency. A 1% increase in the level of diversified and specialized agglomeration modes enhances the green development efficiency by 12.8% and 6.2%, respectively. These results are closely related to the economic characteristics and modern service industry development strategies in eastern China. The cities in this region have a high level of producer services agglomeration. The specialized agglomeration mode, supported by regional policies and resource endowments, allows economically developed cities to leverage local advantages, thereby notably enhancing urban green development efficiency. This innovative output of diversified agglomeration relies on robust foundational conditions and a well-established industrial chain, requiring tacit cooperation among industries. The relatively perfect industrial chain in eastern China creates a demanding market for the diversified agglomeration of producer services, thereby continuously enhancing the urban green development efficiency. In contrast, in eastern China, the indirect impact of producer services specialization and diversified agglomeration on urban green development efficiency is pronounced. Notably, the positive effect of diversified agglomeration on urban green development efficiency exceeds that of specialized agglomeration. The measurement results indicate that a 1% increase in the level of professional and diversified agglomeration in the eastern region will lead to a 6.8% and 8.3% increase in the green development efficiency of the surrounding cities, respectively. This outcome could be attributed to the continuous acceleration of industrial structure optimization and upgrading in the eastern region. Additionally, the high-tech service industry, exemplified by producer services, has notably facilitated interregional exchanges, cooperation, and improved resource utilization efficiency.

In the central and western regions, the influence of producer services agglomeration on urban green development efficiency is mainly through professional agglomeration. Conversely, the diversification of producer services agglomeration has a minimal promoting effect on enhancing urban green development efficiency. Specifically, a 1% increase in the level of diversified agglomeration of producer services in the central and western regions leads to a 2.7% and 10.9%, increase in green development efficiency of local cities, respectively, and a 6.2% and 7.1% increase in the green development efficiency of neighboring cities, respectively. However, the indirect effect of the diversified agglomeration of producer services on the efficiency of urban green development efficiency in central and western China only passes the 5% significance test of the model. This phenomenon may be attributed to the fact that many cities in western China rely on different single manufacturing sectors, which typically comprise only a few subdivision industries with relatively simple structures. Consequently, these cities tend to benefit from specialized agglomeration of producer services rather than diversified agglomeration. Specifically, enterprises within the same industry agglomeration in a specific geographic area realize the collection of expertise, technology, information, and skilled individuals within the industry. This collection leads to a synergistic effect, enhancing the economic output efficiency in inland cities. Conversely, if cities in central and western regions blindly promote diversified agglomeration of producer services, they may stray from their inherent regional strengths, potentially hindering the enhancement of green development levels in neighboring cities.

#### Decomposition of spatial effects based on the differences in economic location

Taking the number of permanent residents at the end of 2018 as the proxy variable of city size, and taking 1 and 3 million as the dividing thresholds, Chinese cities are divided into three levels: big cities (>3 million), medium-sized cities (1–3 million) and small cities (<1 million). On this basis, regression is performed in accordance with different city sizes (Table 6).

**Table 6:** Influence of producer services agglomeration on UGDE and spatial spillover effect: City size difference.

Effect type	Variable	Big sized city		Medium-sized city		Small city	
		(1)	(2)	(1)	(2)	(1)	(2)
Direct effect	lnMar	0.039**		0.051***		0.027*	
		2.605		7.659		1.251	
	lnJac		0.051**		0.068***		0.027*
			2.729		7.937		1.595
	lnPre	0.028**	0.038*	0.039**	0.012*	0.046	0.051
		2.395	2.620	2.558	1.779	0.754	0.930
	lnEQ	-0.019**	0.046	0.062*	-0.030	-0.058	0.049
		-2.133	0.835	1.572	-0.617	-0.830	0.758
	lnRGDP	0.084***	0.013**	0.042**	0.095***	0.022**	-0.065
		8.527	2.309	2.830	10.712	2.351	-0.928
	lnTE	0.052**	0.037*	0.041**	0.058***	0.026*	0.065**
		2.709	1.795	2.747	7.042	1.429	2.937
	lnHum	0.017*	-0.036	0.022*	0.058*	0.048**	0.031**
		2.132	-0.805	1.395	1.605	2.636	2.702
Indirect effect	lnMar	0.075***		0.050**		0.047*	
		5.419		2.208		1.305	
	lnJac		0.081***		0.053**		0.062*
			6.902		2.860		1.109
	lnPre	0.031*	0.049	-0.028*	0.025	0.069*	0.057
		1.552	0.793	-0.584	0.603	1.682	0.856
	lnEQ	-0.021	0.014*	-0.037	-0.037*	0.053	0.028**
		-0.592	1.208	-0.709	-1.508	0.716	2.685
	lnRGDP	0.057*	0.036	0.043	0.080**	0.024**	0.072***
		1.785	0.680	0.816	2.673	2.682	6.503
	lnTE	0.063***	0.032***	0.051**	0.047***	0.059**	0.022***
		5.904	4.206	2.837	6.830	2.795	5.158
	lnHum	0.037*	-0.026*	0.049**	0.045	0.051*	-0.030*
		1.665	-0.594	2.927	0.550	1.680	-1.705

Note:

* means p < 0.10,

** means p < 0.05,

*** means p < 0.01.

From the perspective of city scale, the agglomeration of producer services in big cities substantially enhances urban green development, with the spillover effect surpassing the direct impact. The measurement results show that for every 1% increase in the level of specialization and diversification of producer services in big sized cities, the indirect promoting effect on the green development efficiency of surrounding cities increase by 7.5% and 8.1%, respectively. This finding is primarily due to the notable driving effect of producer services in big sized cities on urban industrial growth, which improves the positive externality of industrial agglomeration and the quality of the urban ecological environment. In addition, the improvement of green efficiency in producer services has a positive spatial spillover effect on neighboring cities. The direct effect of producer services agglomeration on urban green development efficiency in medium-sized cities is notably greater than the spatial spillover effect. Measurement results indicate that specialization and diversified agglomeration of producer services have a notable direct impact on urban green development efficiency in these cities. Specifically, each 1% increase in the level of specialization and diversification in producer services results in a 5.1% and 6.8% increase in urban green development efficiency, respectively. This result is mainly due to the substantial pressure on medium-sized cities to transform and upgrade. The challenges of balancing traditional industrial transformation with the introduction and cultivation of advanced industries contribute to the limited spatial spillover effects of producer services agglomeration. On the one hand, during the process of economic transformation, medium-sized cities face the dual challenge of enhancing their economic and industrial structures to address global economic changes and local economic development needs. On the other hand, when attempting to attract high-tech and advanced industries, these cities often struggle to attract high-tech and advanced industries due to insufficient appeal and supporting resources, which in turn, impede the rapid establishment and growth of such industries. Consequently, the development level and vitality of the production-oriented service industry in medium-sized cities are relatively low, and their impact on surrounding areas is weak. In contrast, the agglomeration of producer services in small cities has a limited effect on urban green development, with neither specialization nor diversification agglomeration showing significant direct or spatial spillover effects. Overall, the disparities in city sizes contribute to "potential differences" in urban green development efficiency. Therefore, addressing and managing these differences within cities of varying sizes is crucial for enhancing the eco-friendly effectiveness of urban areas in China.

## Conclusions and discussions

### Conclusions

This paper uses the SBM–Undesirable model to measure and analyze the urban green development efficiency of 284 cities at or above the prefecture level in China from 2002 to 2018. On this basis, the spatial panel Durbin model is used to reveal the spatial effect of producer service agglomeration on urban green development efficiency. The research indicates a clear decline in the specialized agglomeration of producer services, with spatial characteristics showing "east higher than west and north higher than south." The degree of diversified agglomeration shows a steady upward trend and exhibits a geographical spatial pattern of “scattered on a large scale and concentrated on a small scale.” The overall green development efficiency of Chinese cities demonstrates a dynamic upward trend, with notable regional variations and a certain spatial correlation. Producer services agglomeration is positively correlated with urban green development efficiency, with diversified and specialized agglomeration showing positive spillover effects. Furthermore, the impact of producer services agglomeration on green development efficiency exhibits remarkable regional differences and industry-specific heterogeneity.

### Discussions

In this paper, producer services agglomeration and urban green development are incorporated into the same research framework. The impact of producer services agglomeration on urban green development efficiency is analyzed from the perspective of agglomeration externality, verifying the theoretical hypothesis that producer services agglomeration is intrinsically linked to the improvement of urban green development efficiency. The dynamic changes in urban green development efficiency can be attributed to the combined effects of natural background and human economic factors, which cannot be fully explained solely from a humanistic or societal perspective [2, 3, 4]. Therefore, this study enriches the theoretical understanding of green development efficiency through three dimensions: nature, society, and economy, providing valuable insights for the overall control and promotion of regional green development. In addition, some scholars argue that China’s economic development exhibits a sustained environmental "EKC" effect [1]. This study confirms the hypothesis and finds that producer services agglomeration has an inhibitory effect on this phenomenon. Therefore, adjusting the relationship between economic and social development and environmental pollution crucial for achieving green development, and the advancement of producer services offers a new way to realize this goal. Previous studies have shown that producer services not only enhance exchanges and cooperation between different local organizations but also influence the industrial development of neighboring areas, thereby promoting regional green development [45, 46]. In light of the existing studies, the conclusions of this paper empirically support the theoretical hypothesis that specialized and diversified agglomeration of producer services have heterogeneous and spillover effects on urban green development efficiency.

Producer services agglomeration is a crucial driver of urban economic growth and green development in China, especially during the transition period. Effectively managing its role in the green development process will have a direct impact on regional sustainable development. Therefore, government departments should carefully consider the different types of producer services agglomeration and guide their formation into a reasonable spatial layout. In addition, modifying the configuration and agglomeration mode of producer services based on the size and geographical position of urban areas is necessary to accelerate regional green transformation and production efficiency improvement. The specific strategies are as follows: First, prioritizing producer services with competitive advantages toward engagement in professional agglomeration is crucial for all regions. This approach will improve regional green development efficiency by increasing technology application in high-tech industries and leveraging the external benefits of professional agglomeration. Meanwhile, fully utilizing the external effects of industrial diversification, such as economy of scale and the diffusion of knowledge and technology, is imperative to enhance the efficiency of regional green development. Second, cities of different sizes should adopt differentiated development modes tailored to their development characteristics. As the core drivers of regional development, big cities should rely on a reasonable agglomeration model of the industry and accelerate the diversified integration and innovation of producer services. In addition, the government must use the spatial spillover impact of the agglomeration of producer services in the eastern region. At present, the development level of producer services with high industrial added value and technological content in the eastern region is notably higher than that in the central and western regions. Therefore, cities located in the eastern area must accelerate the cultivation and development of advanced producer services. This acceleration will not only enhance the spatial spillover to cities in the central and western regions but also promote the green development of cities in the central and northwest regions through the trans-regional output of producer services.

## Supporting information

S1 Data (ZIP)
